# Antioxidation Effect of Simvastatin in Aorta and Hippocampus: A Rabbit Model Fed High-Cholesterol Diet

**DOI:** 10.1155/2016/6929306

**Published:** 2015-12-20

**Authors:** Guangyin Zhang, Ming Li, Yinzhi Xu, Li Peng, Cui Yang, Yanan Zhou, Junping Zhang

**Affiliations:** ^1^Department of Psychosomatic, First Teaching Hospital of Tianjin University of TCM, Tianjin 300193, China; ^2^Department of Cardiology, First Teaching Hospital of Tianjin University of TCM, Tianjin 300193, China; ^3^Department of Geriatrics, First Teaching Hospital of Tianjin University of TCM, Tianjin 300193, China; ^4^Tianjin University of Traditional Chinese Medicine, Tianjin 300193, China; ^5^First Teaching Hospital of Tianjin University of TCM, Room 309, Office Building, No. 314, An Shan Xi Road, Nankai District, Tianjin 300193, China

## Abstract

We show that hypercholesterolemia contributes to oxidative stress injury progression in brain and simvastatin counteracts the cholesterol-induced peroxidation injury in rabbit hippocampus, and we demonstrate for the first time that the simvastatin is a critical role in brain protection and identify HO-1 and other related antioxidant enzymes as molecular target for active redox compounds. Second, our experiments have pointed out an association between statin treatment and a decrease in the risk of having peroxidation damage of brain. The balance effects of simvastatin to ROS and antioxidants enzymes network are most probably due to improved SOD functional activity, increase in GSH-Px, increase in HO-1 expression, and decrease of MDA generation.

## 1. Introduction

Hypercholesterolemia is a major risk factor for age-related diseases such as atherosclerosis, obesity [1], and cardiovascular disease [2], and it has been known that the risk factors of various brain diseases also play part in cardiovascular diseases such as dementia, including its most common form, Alzheimer's disease (AD) [3]. The hippocampus is a major component of the brains of humans, which is exceptionally susceptible to oxidative stress that may be caused by hypercholesterolemia [4]. During the past few years, more evidences have accumulated that high-cholesterol level may increase the risk of developing dementia in the action of lipid metabolism-related enzymes [5] and oxidative stress-related proteins [6]. To our knowledge, hypercholesterolemia is known to be sufficient to promote metabolic dysfunction [7], but little is known about its effect on hippocampus.

Hypercholesterolemia is believed to cause oxidative stress by increasing the production of reactive oxygen species (ROS), and which play an important role in neuronal cell death, which is associated with many different neurodegenerative conditions, also is a key event in a variety of inflammatory processes [8, 9]. Recently, many studies have reported that memory impairment in the hypercholesterolemia-induced animal model seems to have correlation with the level of oxidative stress within the brain [10, 11]. Heme oxygenase-1 (HO-1), also referred to as HSP32, belongs to the HSP family and protects mammalian cells from oxidative stress by degrading toxic heme into free iron, carbon monoxide, and biliverdin/bilirubin. The enzyme HO-1 functions as an antioxidant and serves to protect against tissue injury, and the inducible form of HO-1 has been recently demonstrated to exacerbate early brain injury produced by hypercholesterolemia [12, 13]. It has been suggested that the accumulation of HO-1 proteins in the brain may be a protective response to oxidative stress [14].

The concentration of plasma cholesterol can be regulated by cholesterol biosynthesis, removal of cholesterol from the circulation, absorption of dietary cholesterol, or excretion of cholesterol via bile and feces. Simvastatin (HMG-CoA reductase inhibitors) and cholesterol-lowering drugs, which are now widely prescribed to patients with ischemic heart diseases, have reported that it does have antioxidant [15], anti-inflammatory [16], and immunomodulatory benefits [17] as well as its therapeutic use in hyperlipidemia [18]. However, the mechanisms underlying these benefits are not yet completely understood. In this study, we detected the lipid peroxidation products and used them as a biomarker of oxidative stress of hippocampus in rabbit's hypercholesterolemia atherosclerotic model. We also evaluated the activity of defensive enzymes, including superoxide dismutase (SOD), glutathione peroxidase (GSH-Px), catalase (CAT), and HO-1, as a marker of oxidative status in the hippocampus.

## 2. Materials and Methods

### 2.1. Subjects, Housing, and General Procedures

All animal experiments were performed with the approval of the Animal Care Committee of the Tianjin University of Traditional Chinese Medicine and complied with the Animal Management Rule of the Ministry of Public Health, People's Republic of China (Documentation number 55, 2001, Ministry of Health of PR China). Twenty-four adult (3 months old, 2.0 ± 0.2 kg), male, Japanese white rabbits were purchased from Vital River Lab Animal Technology Co., Ltd. (Beijing, China) and were housed in an animal room maintained at 22 ± 2°C with 40% to 60% RH and a light period from 8:00 to 20:00 in the Laboratory Animal Center of Tianjin university of TCM. All the animals had free access to water. Cleaning of the cages and weighing of the rabbits were conducted once every two weeks. After 1 week of habituation and acclimatization, the experimental procedures were started.

### 2.2. Experimental Design

The rabbits were divided randomly into two groups: normal diet group (control, *n* = 8) were fed the rabbit standard diet (100 g per rabbit per day) and experimental model group (*n* = 16) were fed an atherogenic diet (1% cholesterol, 5% yolk, 5% lard, and 89% standard diet, 100 g per rabbit per day). The robust rabbit model of atherosclerosis was generated by the methods established in our laboratory as reported previously [19]. After 8 weeks on high-cholesterol diet, the experimental model group animals were divided into two groups: high-cholesterol diet group (HCD, *n* = 8) and Simvastatin group (HCD + Simvastatin, *n* = 8). All rabbits were maintained on these respective diets and Simvastatin treatments for 16 weeks and all the rabbits were euthanized.

### 2.3. Collection of Blood Samples and Isolation of Hippocampus

The animals were euthanized with an overdose of sodium pentobarbital. Animals' blood was taken through cardiac puncture at the end of week 24 (30 mL each rabbit). Blood samples were centrifuged at 3 500 rpm for 20 minutes to obtain serum and plasma. The collected serum and plasma in aliquots were stored at −80°C until day of assay. The hippocampus was rapidly excised using a stainless steel surgical apparatus from the brain and kept frozen quickly at temperature −80°C until being analyzed. Half of the brain was taken for Hematoxylin and Eosin (H&E) staining and immunohistochemistry staining from the other half the section was dissected for enzyme linked immunosorbent assay (ELISA) and quantitative real time RT-PCR (qRT-PCR).

### 2.4. Enzyme Linked Immunosorbent Assay

The hippocampus was rapidly chipped out about 100 mg and homogenized with tissue protein extraction reagent (CWBIO, Beijing, China) containing protease inhibitors. The homogenates were centrifuged at 10,000 ×g at 4°C for 10 min. The supernatants were collected and used for the ELISA. For the assay, the rabbit SOD, MDA, and GSH-Px immunoassay kits (catalog CSB-E137 11Rb, CSB-E13712Rb, and CSB-E13970Rb; Cusabio Biotech Co., California, USA) were used. The expressions of SOD and MDA were measured in homogenate of hippocampus. Levels of SOD, MDA, and GSH-Px in serum were also measured. Every sample was assayed in duplicate. The absorbance was measured at 450 nm by an ELISA plate reader (Thermo MK3, USA), and all measurements were performed according to the manufacturer's protocol.

### 2.5. Hematoxylin and Eosin Staining

To determine the presence of peroxidative damage and identify pathological changes in the rabbit hippocampus, frozen hippocampal sections were collected and performed for H&E staining. After taking out the freezing hippocampus from ultracold storage (−80°C), coronal sections (40 *μ*m thick) were cut on a cryostat (Leica CM3050, Germany) at −20°C. Perfused sections were mounted onto poly-L-lysine coated slides; the fresh frozen sections were thawed, dried, postfixed in 70% ethanol overnight prior to staining, and then conventionally stained with H&E.

### 2.6. Immunohistological Analysis

Fresh frozen sections were thawed, dried, and then immersed in precooled acetone (−20°C) for 10 min. Endogenous peroxidase activity was blocked by 3% (v/v) H_2_O_2_, and the antigen was retrieved by microwave in 0.01 mol/L citrate buffer. Sections were then washed in PBS (0.1 mol/L). Goat polyclonal anti-HO-1 (Santa Cruz Biotechnology, CA, USA) and mouse anti-goat (Santa Cruz Biotechnology, CA, USA) occluding secondary antibodies were applied at 1 : 100 and incubated overnight at 4°C. Sections were washed four times in PBS for 20 min. All slides were analyzed blindly with respect to treatment condition, using an Olympus CKX31 light microscope (Olympus America Inc., Tokyo, Japan).

### 2.7. Quantitative Real Time Reverse Transcriptase-PCR Analysis

The hippocampus was immediately sonicated in GTC lysis buffer (4 M GTC, 25 mM tribasic sodium citrate, 100 mM TRIS, pH 7.5, containing 0.5% N-lauroylsarcosine, and 0.8%  *β*-mercaptoethanol). Total RNA was extracted with a purification kit (RNeasy mini kit; Qiagen, Valencia, CA) according to the manufacturer's instructions. The amount of total RNA was quantified with a photometer (BioPhotometer Plus; Eppendorf, Germany). For reverse transcription, 2 *μ*g total RNA was combined with 50 nM Oligo (dT)18 primer using concentrations for reaction. Single-strand cDNA was synthesized from the RNA by adding the following reagents (final concentrations): 5x first-strand buffer, 40 U/*μ*L RNAsin, 10 *μ*M each dNTP, and 200 UM-MLV reverse transcriptase (M1701, Promega, Madison, USA). The reaction mixture (40 *μ*L) was incubated at 42°C for 60 minutes, and heating the mixture to 95°C for 5 minutes terminated the reaction. The samples were stored at −20°C for PCR analysis.

Quantitative RT-PCR was carried out on a PCR amplification system and detection system (Rotor-Gene RG-3000, Corbett Robotics Pty Ltd., Australia). Both SYBR Green PCR Kit (204143, QuantiTect) and Go Taq Green Master mix (M7123, Promega, Madison, USA) were used according to the manufacturer's instructions. Expression of target genes was measured in triplicate and was normalized to *β*-actin in hippocampus and GAPDH in aorta as an internal control. PCR amplification of HO-1 mRNA was carried out with the following primers: 5′TGC CGA GGG TTT TAA GCT GGT 3′ (forward) and 5′AGA AGG CCA TGT CCA GCT CCA 3′ (reverse). The mRNA of *β*-actin served as the loading control. Gene expression was quantified using a modification of the 2^−ΔΔCt^ method, as previously described [20].

### 2.8. Data Analysis

The Statistical Package for the PASW Statistics 18.0 was used for the analysis. Results are given as mean ± SD. Data were analyzed statistically using one-way-ANOVA test followed by LSD posttest and pairwise multiple comparisons were performed using LSD posttest. In all instances, *P* value less than 0.05 or 0.01 was considered significant.

## 3. Results

### 3.1. Simvastatin Therapy Enhanced HO-1 in Hippocampus and Also in Aorta

**Figure 1:**
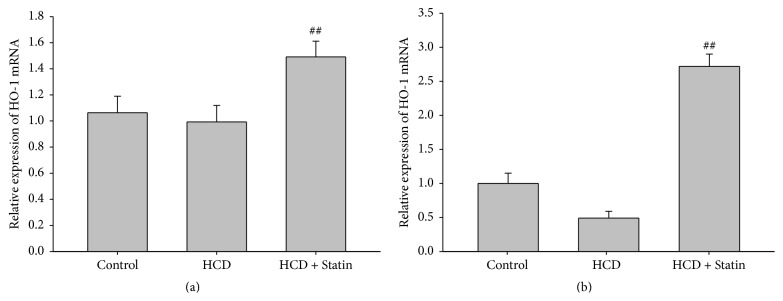
Simvastatin therapy enhanced HO-1 mRNA in hippocampus and also in aorta. (a) HO-1 mRNA expression in aorta; (b) levels of HO-1 mRNA in rabbit hippocampus homogenate. Data are presented as mean ± SD (*n* = 8). ## means *P* < 0.01 indicates significant change from control groups, at *P* < 0.01 using ANOVA followed by LSD as a one-ANOVA test.

It is well known that HO-1 plays a crucial role in the oxidative stress process during human atherosclerotic lesions; the HO-1 mRNA and protein expression can be found in various human organs including brain, liver, lung, heart, and kidney [21–23]. To investigate the antiatherogenic effect of statins in the rabbit aorta and brain after aortic balloon injury and high-cholesterol diet, the mRNA level of HO-1 was detected in rabbit aorta and hippocampus using real time fluorescent quantitative PCR (FQ-PCR). As shown in Figure 1(a), HO-1 mRNA expression of high-cholesterol diet group decreased, respectively, in hippocampus, but it rose after treatment with Simvastatin, and rabbit aorta showed the same tendency but not so markedly (Figure 1(b)).

**Figure 2:**
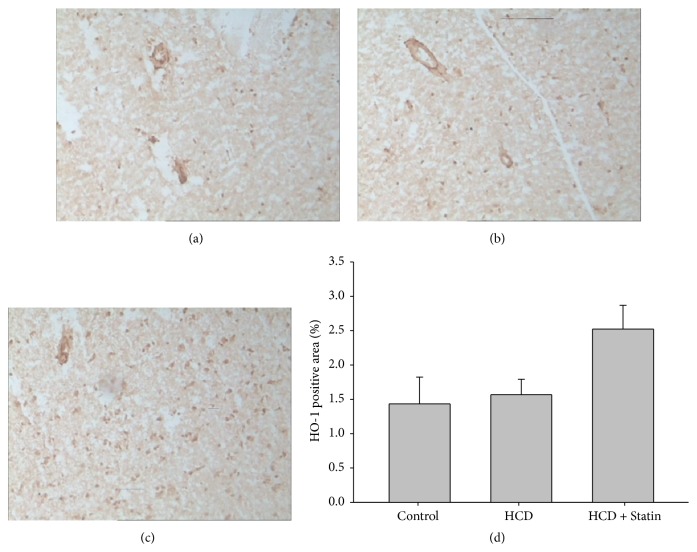
Representative immunohistochemical staining (100x) analysis of rabbit tissues exposed to specific anti-HO-1. (a) Weak staining on the sections of hippocampus from control rabbit. (b) A fewer brown particles in HCD rabbit sections. (c) More brown particles scattered and strong positive staining was observed in the Simvastatin treatment group. (d) HO-1 positive area (%) shown; data are expressed as mean ± SD (*n* = 8), and there was no significant difference in staining degree between each group.

Using immunohistochemical analyses, we further aimed to determine the localization of HO-1 protein in rabbit hippocampus of each group. In a variety of those tissues, brown particles scattered and more immunoreactivity was observed in the Simvastatin treatment group (Figure 2(c)), while staining levels were moderate in HCD rabbit tissues (Figure 2(b)) and there were low levels of immunohistochemical staining in normal rabbit (Figure 2(a)). HO-1 positive area was detected in the hippocampus slices, but there was no significant difference in staining degree between the three groups; however, comparing to HCD group, the HO-1 positive areas in statin treatment group were obviously, as shown in Figure 2(d) (*P* = 0.052). Additionally, HO-1 protein immunoreactivity was observed in capillary endothelial cells, which represent the statin induced HO-1 in endothelial cells and reducing peroxidation and preventing brain-capillary barrier dysfunction [24].

### 3.2. Atherogenic Diet Increased Oxidative Stress and Antioxidant Enzymes Imbalance in the Rabbit Brains

**Figure 3:**
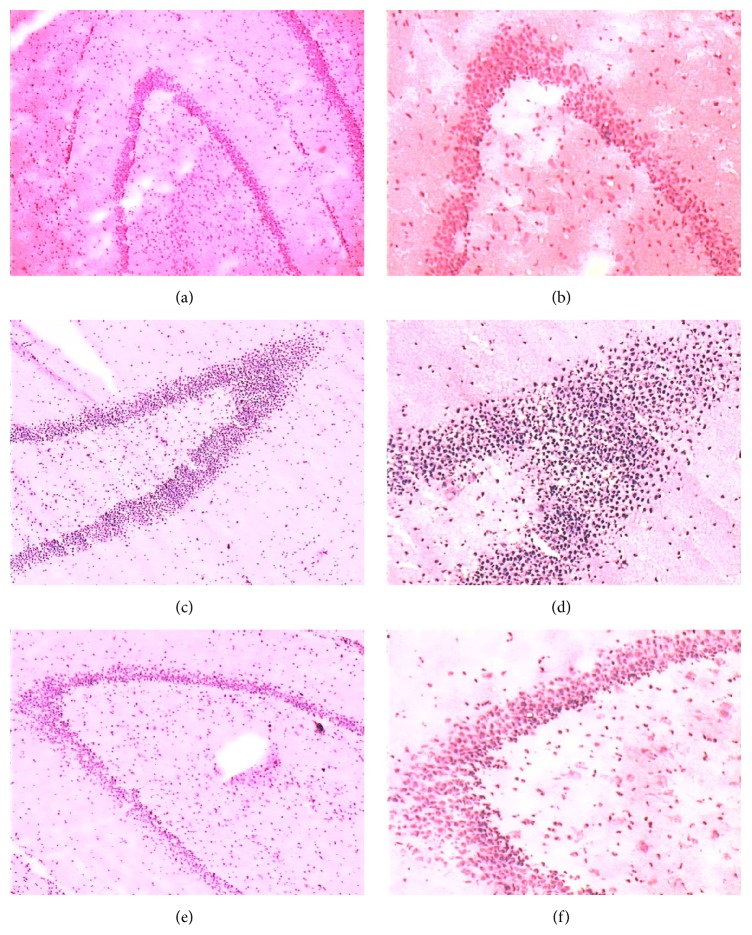
H&E staining of hippocampal sections isolated from rabbit hippocampi. Pyramidal neurons in hippocampus showed no change in H&E staining in the control group. In contrast, atherogenic diet induced a fraction of pyramidal neurons that exhibited morphologic features of necrosis, and there had been fewer necrotic pyramidal neurons than matched sections from Simvastatin treatment group rabbits.

Hematoxylin and Eosin stained sections of hippocampus were examined for signs of peroxidation injury. Animals on the normal diet had no gross pathological changes (Figures 3(a) and 3(b)), and the neurons in hippocampus lined up orderly and compactly, whereas atherogenic diet induced a fraction of pyramidal neurons that exhibited morphologic features of necrosis (Figures 3(c) and 3(d)), which showed triangle, cytoplasm condensation, cellular nucleus pyknosis, and vacuole, a small number of nucleoli disappeared, intercellular gap enlarged, and most neurons were in a necrotic state, but Simvastatin treatment group had no significant changes comparing to atherogenic diet (Figures 3(e) and 3(f)).

**Figure 4:**
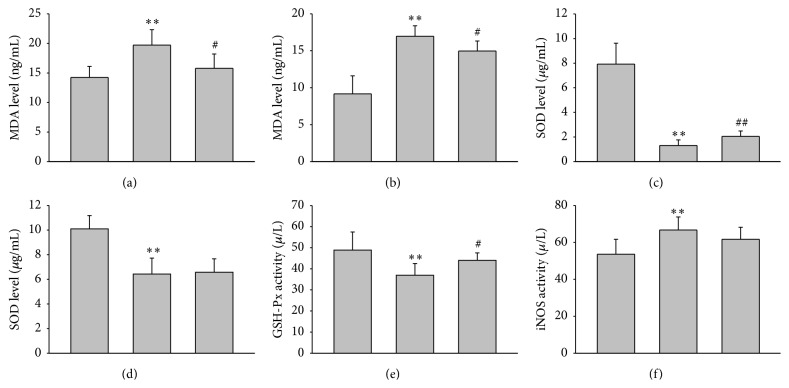
Analysis of biochemical measurements in each group of rabbits; Simvastatin enhanced the antioxidant status in hippocampus and aorta and reduced oxidative stress. (a) Effects of Simvastatin on MDA in serum. (b) Levels of MDA in hippocampal tissues; (c) SOD in serum; (d) levels of SOD in hippocampal homogenate. (e) Activity of GSH-Px in rabbit hippocampus. (f) Activity of iNOS showed in hippocampal tissues. Data are presented as mean ± SD (*n* = 8). *∗∗* and ## indicate significant change from control and HCD groups, respectively, at *P* < 0.01; # means *P* < 0.05 compared to the HCD group using ANOVA followed by LSD as a one-ANOVA test.

The levels of MDA and iNOS were also used to detect damage in serum and hippocampus tissues that GSH-Px and SOD immunoreactivities were used to determine oxidative stress. The figure shows that MDA concentration increased significantly in the serum (Figure 4(a)) and hippocampus (Figure 4(b)) tissues of HCD atherosclerotic rabbit group, compared with the basal level in the control group; activity of iNOS showed the same tendency in rabbit hippocampus, whereas there is a marked decrease in GSH-Px activity in the nontreated atherosclerotic group compared with the control group (Figure 4(e)). Compared to the control group, serum (Figure 4(c)) and hippocampus (Figure 4(d)) homogenate levels of SOD were reduced in the cholesterol diet rabbit group. Interestingly, comparing the two control groups, SOD level in serum was higher than those in hippocampus homogenate.

### 3.3. Simvastatin Enhanced the Antioxidant Status in Hippocampus and Reduced Oxidative Stress

The previous data suggested that statins enhance the antioxidant status likely by inhibiting oxidative stress [4, 18]. Our experiment reveals that there was a marked decrease in GSH-Px expression in the atherosclerotic group compared with the control group; however, treatment of the atherosclerotic group with statins would partly reverse this decline (Figure 4(e)). The MDA levels in aorta would drop obviously after treatment of the atherosclerotic group with statins and also in hippocampus (Figure 4(b)). Compared to the atherosclerotic group, the levels of SOD in serum (Figure 4(c)) and hippocampus homogenate (Figure 4(d)) were increased in the Simvastatin group (Figure 4(d)). However, comparing to HCD group, there was no significant difference in rabbit hippocampus in statin treatment group.

## 4. Discussion and Conclusion

Atherosclerosis is considered to be a systemic vascular inflammatory disease [25], and oxidative stress is known to be a contributor to vascular inflammation [26]. Statins dramatically reduced cardiovascular events in patients with normal lipid levels, but with rarely appraise from oxidative stress levels independently to lipid-lowering properties [27]. In the present study, we show that hypercholesterolemia contributes to oxidative stress injury progression in aorta and brain and Simvastatin counteracts the cholesterol-induced peroxidation injury in rabbit hippocampus, and we demonstrate for the first time that Simvastatin has a critical role in brain protection and identifying HO-1 and other related antioxidant enzymes as molecular target for active redox compounds.

Oxidative stress is a general term used to describe the steady state level of oxidative damage in a cell, tissue, or organ, caused by a dense, complex, and heterogeneous network of oxidizing reactions running counter to the reducing conditions that otherwise prevail in cells and tissues [11, 26]. The experiments were performed using experimental aortic balloon injury and high-cholesterol diet induced rabbits to research the relationship between serum lipoproteins and brain oxidative stress injury and also investigate the antioxidation effect of Simvastatin on hippocampus. The results from our research indicate that HO-1 protein and mRNA were clearly expressed, albeit there is not a statistically significant difference; there still might be a real difference between the control and HCD groups. Comparing with control group, the atherosclerotic rabbits may be a process of adaptation to hypercholesterolemia. This is in accordance with other studies, which showed decreased expression of the HO-1 gene in atherosclerotic rabbits [28], as well as in the ApoE^−/−^ mouse model of atherosclerosis [29].

The brain is a highly metabolically active organ producing large amounts of reactive oxygen species (ROS). These ROS and antioxidants enzymes network are kept in balance anytime [30, 31]. Based on our experiments, we propose that chronic cholesterol exposure damages imbalance in antioxidant enzymes and increased oxidative stress in the brains. The imbalance of ROS and antioxidants enzymes network could lead to neuronal oxidative stress [32], those imbalances associated with not only aging [33] but also Alzheimer's disease [32]. A previous study showed that hypercholesterolemia accelerates intraneuronal accumulation of A*β* oligomers and subsequent synapse loss in Alzheimer's disease model mice [34]. Our experiments show that MDA concentration increased significantly in the serum and also on hippocampus tissues of HCD atherosclerotic rabbit group; Hematoxylin and Eosin stained slices of hippocampus showed triangle, cytoplasm condensation, cellular nucleus pyknosis, and vacuole, a number of nucleoli disappeared, intercellular gap enlarged, and most neurons were in a necrotic state in atherosclerotic rabbit. Induction of the high-output iNOS usually occurs in an oxidative environment [35], which were bright enough to confirm our experiments.

A large number of experimental observations have suggested that Simvastatin is a useful treatment in encephalopathy like vascular dementia [36] and Alzheimer's disease [37] with dyslipidemia and an atherogenic lipid profile characterized by alterations in cholesterol homeostasis. This is a pleiotropic statin effect that is independent of lipid lowering [38, 39]. It has already been suggested that induction of HO-1 during atherosclerosis might ameliorate oxidative stress [40] and represent an adaptive response to hypercholesterolemia, and other studies indicate that statins could influence HO-1 regulation [41] and superoxide production on primary cultures of cortical neurons on neuro-2A cells [42]. From this study, a mild decrease in HO-1 was observed in atherosclerotic animals brain but markedly increased in case of treatment with Simvastatin. To GSH-Px, there was a marked decrease in GSH-Px expression in the atherosclerotic group and treatment of the atherosclerotic rabbit with Simvastatin partly reversed this decrease; maybe it provided more protection to the brain, but a previous experiment shows that atorvastatin did not reverse the cognitive impairments and failed to alter the hippocampal glutamate uptake in A*β*-treated mice [43]. In our experiments, Simvastatin could increase the level of SOD in serum but failed to ameliorate the SOD on hippocampus. Hsieh et al. showed differential induction of antioxidant enzyme by different statins and showed the different physiological relevance of experiments [24]. Our experiments have pointed out an association between statin treatment and a decrease in the risk of having peroxidation damage of brain. The balance effects of Simvastatin on ROS and antioxidants enzymes network are most probably due to improved SOD functional activity, increase in GSH-Px, increase in HO-1 expression, and decrease of MDA generation.

Our previous studies developed a modified protocol of the JW rabbit that reproduced features of atherothrombosis observed in humans [19] and have revealed that oxidative stress plays a crucial role in these rabbits. We used this model to investigate the antioxidation effect of Simvastatin in rabbit aorta and hippocampus and found that the attenuation of atherosclerotic lesions in statin treated group was independent of lipid-lowering function. These findings may be consistent with some researches of antioxidant effects of statin recently [38, 39, 44] and further studies are needed for verifying.

## References

[1] Veghari G., Sedaghat M., Maghsodlo S. (2015). The association between abdominal obesity and serum cholesterol level. *International Journal of Applied and Basic Medical Research*.

[2] De Winter C. F., Bastiaanse L. P., Hilgenkamp T. I. M., Evenhuis H. M., Echteld M. A. (2012). Cardiovascular risk factors (diabetes, hypertension, hypercholesterolemia and metabolic syndrome) in older people with intellectual disability: results of the HA-ID study. *Research in Developmental Disabilities*.

[3] Dias I. H. K., Polidori M. C., Griffiths H. R. (2014). Hypercholesterolaemia-induced oxidative stress at the blood-brain barrier. *Biochemical Society Transactions*.

[4] Métais C., Hughes B., Herron C. E. (2015). Simvastatin increases excitability in the hippocampus via a PI3 kinase-dependent mechanism. *Neuroscience*.

[5] Cheng Y., Jin Y., Unverzagt F. W. (2014). The relationship between cholesterol and cognitive function is homocysteine-dependent. *Clinical Interventions in Aging*.

[6] Chiapinotto Spiazzi C., Bucco Soares M., Pinto Izaguirry A. (2015). Selenofuranoside ameliorates memory loss in Alzheimer-like sporadic dementia: AChE activity, oxidative stress, and inflammation involvement. *Oxidative Medicine and Cellular Longevity*.

[7] Aguilar D., Fernandez M. L. (2014). Hypercholesterolemia induces adipose dysfunction in conditions of obesity and nonobesity. *Advances in Nutrition*.

[8] Viecili P. R. N., Borges D. O., Kirsten K. (2014). Effects of *Campomanesia xanthocarpa* on inflammatory processes, oxidative stress, endothelial dysfunction and lipid biomarkers in hypercholesterolemic individuals. *Atherosclerosis*.

[9] Yi C.-X., Al-Massadi O., Donelan E. (2012). Exercise protects against high-fat diet-induced hypothalamic inflammation. *Physiology and Behavior*.

[10] Zhang L., Ya B., Yang P. (2014). Impact of carotid atherosclerosis combined with hypercholesterolemia on cerebral microvessels and brain parenchyma in a new complex rat model. *Neurochemical Research*.

[11] Axelsen P. H., Komatsu H., Murray I. V. J. (2011). Oxidative stress and cell membranes in the pathogenesis of Alzheimer's disease. *Physiology*.

[12] Lin S. H., Song W., Cressatti M., Zukor H., Wang E., Schipper H. M. (2015). Heme oxygenase-1 modulates microRNA expression in cultured astroglia: implications for chronic brain disorders. *Glia*.

[13] Yoo S., Nakra N. K., Ronnett G. V., Moon C. (2014). Protective effects of inducible HO-1 on oxygen toxicity in rat brain endothelial microvessel cells. *Endocrinology and Metabolism*.

[14] Zeng Z., Huang H.-F., He F., Wu L.-X., Lin J., Chen M.-Q. (2012). Diazoxide attenuates ischemia/reperfusion injury via upregulation of heme oxygenase-1 after liver transplantation in rats. *World Journal of Gastroenterology*.

[15] Wang K.-W., Wang H.-K., Chen H.-J. (2014). Simvastatin combined with antioxidant attenuates the cerebral vascular endothelial inflammatory response in a rat traumatic brain injury. *BioMed Research International*.

[16] Stein A., Stroobants S., Gieselmann V., D'Hooge R., Matzner U. (2015). Anti-inflammatory therapy with simvastatin improves neuroinflammation and CNS function in a mouse model of metachromatic leukodystrophy. *Molecular Therapy*.

[17] Zhu J., Gao B. (2015). Simvastatin combined with aspirin increases the survival time of heart allograft by activating CD4^+^CD25^+^ Treg cells and enhancing vascular endothelial cell protection. *Cardiovascular Pathology*.

[18] Jose M. A., Anandkumar S., Narmadha M. P., Sandeep M. (2012). A comparative effect of atorvastatin with other statins in patients of hyperlipidemia. *Indian Journal of Pharmacology*.

[19] Zhang G., Li M., Li L. (2012). The immunologic injury composite with balloon injury leads to dyslipidemia: a robust rabbit model of human atherosclerosis and vulnerable plaque. *Journal of Biomedicine and Biotechnology*.

[20] Livak K. J., Schmittgen T. D. (2001). Analysis of relative gene expression data using real-time quantitative PCR and the 2(-Delta Delta C (T)) method. *Methods*.

[21] Bakhautdin B., Das D., Mandal P. (2014). Protective role of HO-1 and carbon monoxide in ethanol-induced hepatocyte cell death and liver injury in mice. *Journal of Hepatology*.

[22] Liu X., Zang P., Han F., Hou N., Sun X. (2015). Renal protective effects of induction of haem oxygenase-1 combined with increased adiponectin on the glomerular vascular endothelial growth factor-nitric oxide axis in obese rats. *Experimental Physiology*.

[23] Fukui H., Horie M., Endoh S. (2012). Association of zinc ion release and oxidative stress induced by intratracheal instillation of ZnO nanoparticles to rat lung. *Chemico-Biological Interactions*.

[24] Hsieh C.-H., Jeng J. C.-Y., Hsieh M.-W. (2011). Involvement of the p38 pathway in the differential induction of heme oxygenase-1 by statins in Neuro-2A cells exposed to lipopolysaccharide. *Drug and Chemical Toxicology*.

[25] Ross R. (1999). Atherosclerosis—an inflammatory disease. *The New England Journal of Medicine*.

[26] Ramana K. V., Srivastava S., Singhal S. S. (2013). Lipid peroxidation products in human health and disease. *Oxidative Medicine and Cellular Longevity*.

[27] Brugts J. J., Yetgin T., Hoeks S. E. (2009). The benefits of statins in people without established cardiovascular disease but with cardiovascular risk factors: meta-analysis of randomised controlled trials. *British Medical Journal*.

[28] Li T., Tian H., Zhao Y. (2011). Heme oxygenase-1 inhibits progression and destabilization of vulnerable plaques in a rabbit model of atherosclerosis. *European Journal of Pharmacology*.

[29] Shen Y., Ward N. C., Hodgson J. M. (2013). Dietary quercetin attenuates oxidant-induced endothelial dysfunction and atherosclerosis in apolipoprotein e knockout mice fed a high-fat diet: a critical role for heme oxygenase-1. *Free Radical Biology and Medicine*.

[30] Shim S.-Y., Kim H.-S. (2013). Oxidative stress and the antioxidant enzyme system in the developing brain. *Korean Journal of Pediatrics*.

[31] Massaad C. A., Klann E. (2011). Reactive oxygen species in the regulation of synaptic plasticity and memory. *Antioxidants and Redox Signaling*.

[32] Lim J. L., Wilhelmus M. M. M., de Vries H. E., Drukarch B., Hoozemans J. J. M., van Horssen J. (2014). Antioxidative defense mechanisms controlled by Nrf2: state-of-the-art and clinical perspectives in neurodegenerative diseases. *Archives of Toxicology*.

[33] Haxaire C., Turpin F. R., Potier B. (2012). Reversal of age-related oxidative stress prevents hippocampal synaptic plasticity deficits by protecting D-serine-dependent NMDA receptor activation. *Aging Cell*.

[34] Matsumura A., Emoto M. C., Suzuki S. (2015). Evaluation of oxidative stress in the brain of a transgenic mouse model of Alzheimer disease by in vivo electron paramagnetic resonance imaging. *Free Radical Biology and Medicine*.

[35] Dhar I., Dhar A., Wu L., Desai K. M. (2012). Arginine attenuates methylglyoxal- and high glucose-induced endothelial dysfunction and oxidative stress by an endothelial nitric-oxide synthase-independent mechanism. *Journal of Pharmacology and Experimental Therapeutics*.

[36] Tong X.-K., Hamel E. (2015). Simvastatin restored vascular reactivity, endothelial function and reduced string vessel pathology in a mouse model of cerebrovascular disease. *Journal of Cerebral Blood Flow and Metabolism*.

[37] Tramontina A. C., Wartchow K. M., Rodrigues L. (2011). The neuroprotective effect of two statins: simvastatin and pravastatin on a streptozotocin-induced model of Alzheimer's disease in rats. *Journal of Neural Transmission*.

[38] Wang Y., Bai L., Lin Y. (2015). Combined use of probucol and cilostazol with atorvastatin attenuates atherosclerosis in moderately hypercholesterolemic rabbits. *Lipids in Health and Disease*.

[39] Tong X.-K., Lecrux C., Hamel E. (2012). Age-dependent rescue by simvastatin of Alzheimer's disease cerebrovascular and memory deficits. *The Journal of Neuroscience*.

[40] Pechlaner R., Willeit P., Summerer M. (2014). Heme oxygenase-1 gene promoter microsatellite polymorphism is associated with progressive atherosclerosis and incident cardiovascular disease. *Arteriosclerosis, Thrombosis, and Vascular Biology*.

[41] Heeba G., Moselhy M. E., Hassan M., Khalifa M., Gryglewski R., Malinski T. (2009). Anti-atherogenic effect of statins: role of nitric oxide, peroxynitrite and haem oxygenase-1. *British Journal of Pharmacology*.

[42] Melegari S. P., Perreault F., Moukha S., Popovic R., Creppy E. E., Matias W. G. (2012). Induction to oxidative stress by saxitoxin investigated through lipid peroxidation in Neuro 2A cells and *Chlamydomonas reinhardtii* alga. *Chemosphere*.

[43] Piermartiri T. C., Figueiredo C. P., Rial D. (2010). Atorvastatin prevents hippocampal cell death, neuroinflammation and oxidative stress following amyloid-*β*
_1–40_ administration in mice: evidence for dissociation between cognitive deficits and neuronal damage. *Experimental Neurology*.

[44] Ma Y., Chen Z., Zou Y., Ge J. (2014). Atorvastatin represses the angiotensin 2-induced oxidative stress and inflammatory response in dendritic cells via the PI3K/Akt/Nrf 2 pathway. *Oxidative Medicine and Cellular Longevity*.

